# Accuracy and reliability of a commercial treatment planning system in nontarget regions in modern prostate radiotherapy

**DOI:** 10.1002/acm2.14003

**Published:** 2023-05-11

**Authors:** Willeke Danckaert, Piet Ost, Carlos De Wagter

**Affiliations:** ^1^ Department of Human Structure and Repair, Faculty of Medicine and Health Sciences Ghent University Ghent Belgium; ^2^ Department of Radiation Oncology Ghent University Hospital Ghent Belgium; ^3^ Department of Radiation Oncology Iridium Netwerk Wilrijk Belgium

**Keywords:** microdiamond detector, nontarget dose accuracy, treatment planning system

## Abstract

**Background:**

The currently available treatment planning systems (TPSs) are neither designed nor intended for accurate dose calculations in nontarget regions. The aim of this work is to quantify the accuracy and reliability of nontarget doses calculated by a commercially available TPS.

**Methods:**

Nontarget doses calculated by the collapsed cone (CC) (v5.2) algorithm implemented in the RayStation (v6) TPS were compared to measured values. Different scenarios were investigated, from simple static fields to intensity modulated radiotherapy (IMRT) and volumetric modulated arc therapy (VMAT) treatment plans. Deviations and confidence limits (CLs) were calculated between results of calculations and measurements—applying both local (δ) and global (Δ) normalization—for various points of interest (POIs). Results were based on a single‐institution experience for one clinical test case (prostate) and evaluated against internationally accepted criteria.

**Results:**

Overall, the TPS underestimated the nontarget dose by an average of −17.7% ± 25.3% for IMRT. Quantitatively similar results were obtained for VMAT (−17.6% ± 21.2%). POIs receiving < 5% of the prescription dose were significantly underestimated by the TPS (*p*‐value < 0.05 for both IMRT and VMAT). Dose calculation accuracy was also determined by the contribution of secondary radiation, with measured doses for out‐of‐field POIs being significantly different from calculated values (*p*‐value < 0.01 for both IMRT and VMAT). Although the CL_δ_ in nontarget regions failed the proposed tolerance criteria (40%) for both IMRT (68.8%) and VMAT (52.6%), the CL_Δ_ was within the tolerance limit (4%) for both treatment techniques (1.9% for IMRT and 1.3% for VMAT). No action levels (7%) were exceeded.

**Conclusions:**

Based on the currently available benchmarks our TPS is considered acceptable for clinical use, although the dose in some POIs was poorly predicted by the CC algorithm. Some areas were pointed out where TPSs and linear accelerator control systems can be improved.

## 1 INTRODUCTION

Photon doses delivered to the primary target(s) during radiotherapy can be rapidly and accurately calculated by the current commercially available treatment planning systems (TPSs). However, some TPSs have shown poor calculation accuracy for doses to normal tissues outside the primary target, often referred to as nontarget dose.^1^ TPSs are neither designed nor intended for accurate nontarget dose calculations due to the limited modeling of scattered radiation. Consequently, these doses are not known with any real precision.^1^ Already in 2001, Cozzi et al. emphasized that an accurate dose calculation in the entire range of dose levels could influence the choice between dose escalation schemes, treatment techniques or treatment plans.^2^ Moreover, dose constraints have been developed to minimize radiation‐induced normal tissue injury, but the establishment of the underlying valid dose‐response relationships requires a reliable determination of the complete dose distribution in the patient.^3^

Multiple studies have investigated the accuracy of TPSs in nontarget regions with some reporting large discrepancies between measured and calculated doses, even when modern calculation algorithms were used.^4^, ^5^, ^6^ Howell et al. found that the Eclipse analytical anisotropic algorithm (AAA) (v8.6) underestimated doses in the range of 3.75–11.25 cm from the field edge by 40%  20% compared to measurements with thermoluminescent dosimeters (TLDs) during 3‐dimensional conformal radiotherapy (3D‐CRT).^4^ Similar results have been reported by Colnot et al. using EBT3 radiochromic films. Both the Eclipse AAA and Acuros TPS underestimated the predicted doses as compared to measurements in 3D‐CRT and volumetric modulated arc therapy (VMAT) treatments, with reported local relative discrepancies of −60.4% for 3D‐CRT and −91.2% for VMAT at 15 cm from the planning target volume (PTV) edge.^5^ According to Toossi et al. the average local discrepancy in out‐of‐field regions between the TiGRT TPS (v1.2) and TLD measurements was 35% during breast radiotherapy with two tangential open fields.^6^

The aim of this work was to quantify the accuracy and reliability of nontarget photon doses calculated by the commercially available TPS RayStation in a clinical setting. To our knowledge, there is no other published work assessing the performance of this specific TPS in nontarget regions. Accurate quantification of these low doses may provide deeper knowledge on their impact on surrounding organs at risk (OARs) such as the immune system, which is already known to modulate the clinical response to ionizing radiation.^7^ However, many of these radiation‐induced immunomodulatory effects are complex and are yet to be fully understood.^7^, ^8^ In addition, enhanced knowledge of dose to critical organs may reduce side effects by deriving more accurate dose constraints, which in turn may significantly improve the patient's quality of life.^9^

## 2 MATERIAL AND METHODS

### 2.1 Phantom

**FIGURE 1:**
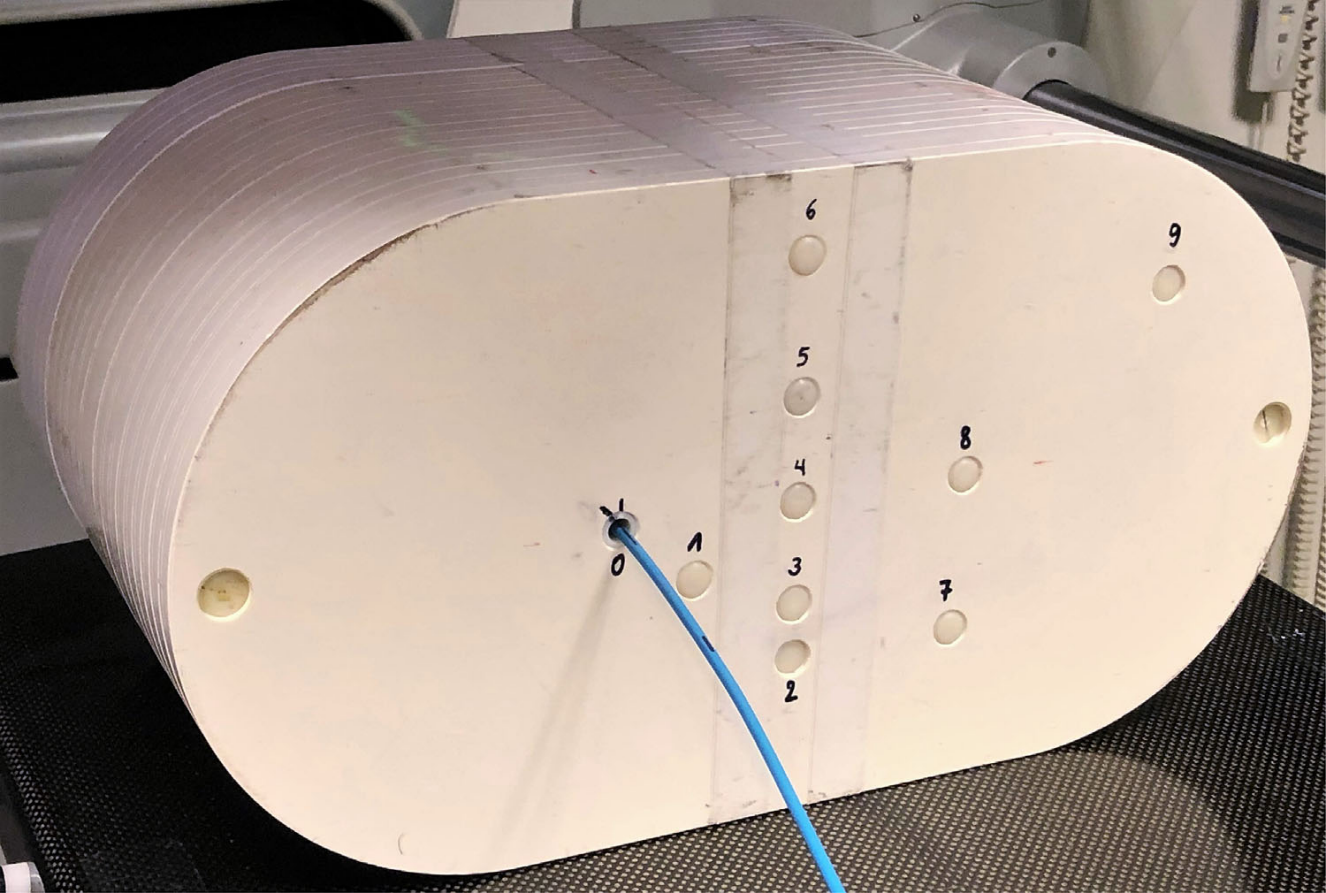
The pelvic polystyrene phantom. Note the measurement holes (0–9) to guide the dosimeter rod, now filled with the dummy rod inserts (1–9). The dosimeter rod is placed in hole 0, with the EPOM of the mD located 8 cm caudally from the isocenter.

All measurements were performed in a pelvic phantom—in essence a longitudinally extended version of the CarPet phantom^10^—composed of 21 identical transverse polystyrene slabs (ρ = 1.02 ± 0.02 g/cm^3^), each with a thickness of 1 cm. The slabs are held together with two clamping screws and ten measurement holes are drilled in the phantom, which allow placement of removable cylindrical rod inserts (length = 23 cm and diameter = 1 cm) made from the same polystyrene material (Figure 1). One rod is hollowed at one end to hold the microDiamond (mD) detector, which is fixed in position with petrolatum (White Vaseline, Qualiphar, Belgium). The effective point of measurement (EPOM) of the detector—actually a 1‐μm thick sensitive volume—is indicated externally with a special reference mark at 11.5 cm from the rod end. Other hatch marks on the rod allow accurate longitudinal placement of the rod insert in the phantom.

### 2.2 Microdiamond detector

All measurements were conducted with a mD detector (PTW 60019, PTW, Freiburg, Germany) (serial number 122204). Variations in the energy spectrum in nontarget regions impose challenges for accurate dose measurements. While scattered radiation has a much softer energy spectrum, head leakage has a comparable spectrum to the primary beam.^11^ The mD detector presents itself as a favorable detector in this context due its low energy dependence (< 1.0%) for photon beam energies between 6 and 18 MV.^12^, ^13^ In addition, the energy response of the mD detector is within 2% for energies above 100 keV.^14^ The low leakage current ≤ ± 20 fA makes the detector suitable for low‐dose measurements.^15^ Previous research also confirmed its excellent dose linearity,^16^, ^17^ dose rate independence,^13^, ^16^, ^17^ and tissue equivalence.^16^ Importantly, an excellent dose response linearity within ± 0.5% was found down to 1.2 cGy for a 2 × 2 cm^2^ field.^17^ Dose rate independence was also below ± 0.5% for dose rates 1−6 Gy/min.^17^ However, the response of the mD can still be biased by a volume‐averaging effect due to the finite area of the sensitive volume, which is basically a micrometer thin 2.2‐mm diameter disk.^18^, ^19^ In addition, this sensitive volume can show local sensitometric non‐uniformities of the order of 15%−30%.^20^ The mD is also prone to overresponding in small radiation fields (< 20 mm),^21^ which may be partly attributed to a radiation‐induced charge imbalance in the metal contacts and cable of the mD detector.^22^ Nevertheless these issues were expected to be insignificant during the experiments. A single point dosimeter was chosen to minimize experimental systematic uncertainties.

The mD detector was always positioned in gun‐target direction in the phantom—which corresponded to the craniocaudal direction in the reference patient—with the sensitive volume facing the gun side in the so‐called “edge‐on” orientation (i.e., with the detector axis parallel to the treatment table axis). The edge‐on orientation^19^ facilitated the access to the aimed measurement points in the phantom with a single detector. During all experiments, the mD was orientated at 0° unless stated otherwise. The detector was pre‐irradiated at isocenter in the phantom before each measuring session.^12^ The mD response was monitored with an electrometer (T10008 Unidos E Universal Dosimeter, PTW, Freiburg, Germany) (serial number 081167) which measured dose by numerical integration. Automatic zeroing was applied when required to the electrometer. The measuring range “Medium” was used. No bias voltage was applied. The average measured isocenter dose across all sessions of a reference field sized 16 × 5 cm^2^ at a collimator angle of 0° was subsequently used to a posteriori correct the electrometer read‐out for intra‐ and intersession linear accelerator (linac) output variations and measurement drift. Per convention, field sizes are defined as A × B with A and B representing the in‐plane and the cross‐plane distance, respectively, with collimator rotation at 0°.

#### 2.2.1 Angular dependence

Given the warning data in literature on the angular dependence of the mD dose response and its criticality in this study, this was to be carefully addressed a priori. To exclude uncertainties due to possible output variations with gantry rotation and in the absence of an ideal spherical phantom, the angular dependence of the mD dose response within its holder was investigated by changing the detector orientation in a static beam (gantry 0°) sized 16 × 5 cm^2^. The detector holding rod was manually rotated in the axial plane with an angular increment of 15° (from 0° to 360°) using an angular scale placed on the phantom. Angular sensitivity was investigated both in‐field (at isocenter and 4 cm cranially from the isocenter) and out‐of‐field (6.5 cm laterally from the isocenter) since its relative magnitude may vary with photon energy.^23^ In addition, to ascertain that nonuniformities in the sensitive volume do not contribute to the angular dependence,^20^ the dose distribution at the level of the mD detector was subsequently made more homogeneous by superposing the doses from two opposing beams (gantry 0° and 180°).

#### 2.2.2 Positioning and centering method

According to the manufacturer, the EPOM in the mD detector is located on the detector axis 1 mm from the detector tip, with its location marked.^15^ However, geometrical manufacturing imperfections in the detector holding rod could have introduced a minor longitudinal shift between the mD's EPOM and its reference mark on the rod. This is a critical concern given the high resolution of the mD in that direction. To experimentally verify the longitudinal isocenter alignment, the isocentric in‐plane profile of a 2 × 10 cm^2^ field (gantry 0°) was acquired by longitudinally moving the treatment couch. The central axis (CAX) deviation in the profile was obtained using the PTW MEPHYSTO mc^2^ software (PTW, Freiburg, Germany) and was used to correct for a possible longitudinal offset of the mD's EPOM.

#### 2.2.3 Cable effect

Attention should also be given to possible spurious signals arising from stem or cable irradiation.^22^ Especially the cable effect may be of importance in nontarget low dose regions, where the dose to the cable may be notably higher than the dose to the detector. The cable effect was quantified by shifting the mD's into a lead cylinder (height = 5 cm and diameter = 10 cm) fixed at the cranial end of the phantom. The induced cable effect could be directly determined as now only the detector cable was basically exposed to radiation. Intensity modulated radiotherapy (IMRT) and VMAT plans were measured in holes 5 and 9 (Figure 1). In these holes, multiple points of interest (POIs) for our clinical prostate plan were located, and the distance from the mD's EPOM to isocenter ranged between 16.7 and 24.4 cm.

### 2.3 Treatment planning and experimental strategy

The polystyrene phantom was scanned with a SOMATOM go.Open Pro (Siemens Healthineers AG, Erlangen, Germany) computed tomography (CT) simulator and images were reconstructed with a 2‐mm slice thickness. In addition, a density override (ρ = 1.02 g/cm^3^) was set on the phantom to disregard slight density differences that arose during the scanning process due to the slabbed configuration of the phantom. Irradiations were performed with an Elekta Synergy (Elekta, Stockholm, Sweden) linac equipped with an Agility™ 160‐leaf multileaf collimator (MLC). Of note, there are no backup jaws in the MLC travel direction. The linac was controlled with the Integrity™ R3.x treatment control system (TCS) (Elekta, Stockholm, Sweden) with treatment plans delivered in quality assurance mode using the MOSAIQ record and verify system (R&V) (v2.82) (Elekta, Stockholm, Sweden). The commercially available TPS RayStation v6 (RaySearch Laboratories AB, Stockholm, Sweden) equipped with a collapsed cone (CC) algorithm (v5.2) was used for dose calculations. The clinically used grid size of 2 × 2 × 2 mm^3^ was applied, unless stated otherwise. Because of the high resolution of the mD in the longitudinal direction, the effect of decreasing the grid size to 1 × 1 × 1 mm^3^ was assessed. All photon beams used in this work had a nominal energy of 6 MV in accordance with the departmental clinical practice^24^ and implying that no beam neutron contamination had to be taken into account.^25^ In order to obtain sufficient precision in the low dose area during the static field experiments (see further), treatment plans were computed with the prescription dose being multiplied by a factor 10. The resulting doses were subsequently divided by the same factor. During all experiments the isocenter was located at insert 0 (depth = 10 cm and source‐to‐surface distance = 90 cm).

#### 2.3.1 Single static field

First, an experimental setup with an unmodulated static field was designed. A rectangular field sized 16 × 5 cm^2^ covering an artificial PTV_a_ of the same dimension was planned on the phantom CT data set, resulting in a nearly constant dose along the longitudinal axis of the mD detector (Figure S1). The field was collimated by both the MLC and orthogonal jaws with gantry and collimator angle set at 0° (MLC_0_). However, this set up would overlook MLC‐jaw combinations that may affect the nontarget doses in a more clinical context. To this end, the MLC was rotated so that its leaves were under 45° to the boundary of the PTV_a_ edges. Experiments were done with both the orthogonal jaws in place (MLC_45_) and fully retracted with jaw openings at 20 × 20 cm^2^ (MLC_45 with jaws retracted_). These setups were considered as an intermediate step towards more complex intensity modulated fields. Thus, three static fields were defined with three different shielding methods with respect to the POIs: (a) MLC_0_; (b) MLC_45_; and (c) MLC_45with jaws retracted_. A total of 22 POIs located outside the primary target (PTV_a_) were selected (Figure S2).

#### 2.3.2 Prostate radiotherapy

**FIGURE 2:**
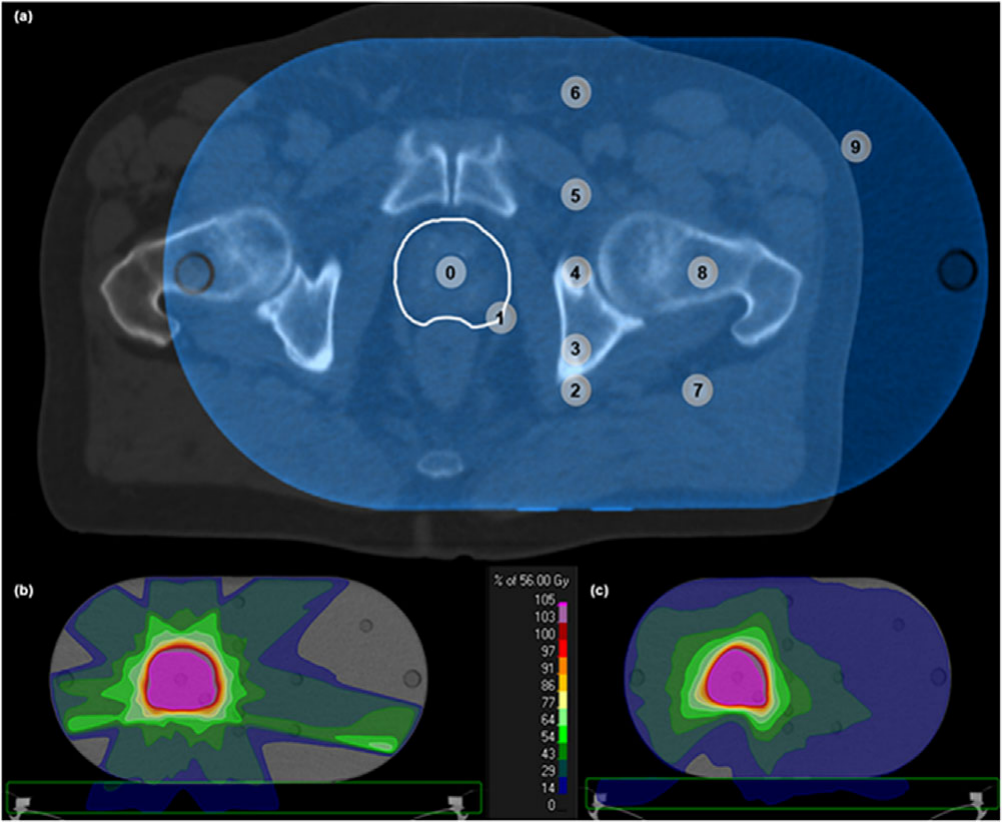
(a) Transverse isocentric plane of the registration of the phantom CT data set (blue) with the patient CT data set (grey). The CTV is shown in white. The grey circles (not to scale) represent the measurement holes with the numbers corresponding to those displayed in Figure 1. (b–c) Transverse dose distribution in the isocentric plane of the (b) IMRT and (c) VMAT prostate treatment plan calculated on the phantom CT data set. The green rectangle represents the treatment table.

To investigate under which circumstances the TPS can calculate nontarget doses with reasonable accuracy and reliability in a clinical context, a reference patient—who was previously treated at our institution with primary radiotherapy for prostate cancer—was retrospectively selected. Ethics approval and written consent were obtained from the local ethics committee (BC‐07902) and patient, respectively. The patient was irradiated with a 2‐arc VMAT plan (from −179° to 180°, clockwise and counterclockwise). MLC rotation angles were 15° and 345°. An equivalent 7‐field dynamic IMRT plan was subsequently created on the same reference patient with gantry angles at 0°, 52°, 103°, 156°, 206°, 257°, and 308°. MLC rotation angles were 0°, 350°, 350°, 350°, 10°, 10°, and 10°, respectively. A median dose of 56 Gy in 16 fractions to the clinical target volume (CTV) was prescribed for both coplanar treatment plans with dose objectives for OARs as previously described.^26^ Both plans were approved by an experienced radiation oncologist, confirming the clinical eligibility. After isocentric registration of the phantom CT data set with the patient CT data set, the clinical treatment plans were recalculated on the phantom CT using the same beam arrangements, monitor units (MUs), and control points (Figure 2). This resulted in a phantom isocenter dose of 60.6 Gy for IMRT and 60.8 Gy for VMAT. A total of 18 POIs were selected, located in measurement holes 5, 7, 8 and 9 (Figure S3) and outside the CTV (except for the isocenter). Each POI was also mapped on the patient CT data set and its anatomical location was registered (Figure S4).

### 2.4 Data analysis and statistical methodology

The calculated dose in POIs along the longitudinal direction of the phantom was compared against the measured dose at the corresponding location. Discrepancies (δ and Δ) between calculations (D_calculated_) and measurements (D_measured_) were defined as percentage dose differences of the locally measured (D_measured_) or prescription dose (D_prescription_):

(1.1) $$\delta \;\left[\%\right]=\frac{D_{\mathrm{calculated}}-D_{\mathrm{measured}}}{D_{\mathrm{measured}}}\;\times \;100\%$$

(1.2) $$\Delta \;\left[\%\right]=\frac{D_{\mathrm{calculated}}-D_{\mathrm{measured}}}{D_{\mathrm{prescription}}}\;\times \;100\%$$

Negative values imply an underestimation of the dose by the TPS. Although local relative dose differences can overstate the clinical importance of the deviation,^27^ normalization using the prescription dose can hide errors in low dose regions.^28^ Thus reported results are for both normalization methods. Of note, 56 Gy was prescribed to the patient's CTV. The computed dose in the phantom isocenter (60.6 Gy for IMRT and 60.8 Gy for VMAT) was used as D_prescription_.

The concept of confidence limit (CL) has been introduced to combine the influence of systematic and random deviations by Venselaar et al.^29^ and was later refined by Palta et al.^30^ The CL was calculated according to Palta et al.^30^

(2.1) $$\begin{array}{c} {\mathrm{CL}}_{\delta }\;\left[\%\right]\;=\;\left|\mathrm{mean}\;\delta \right|\;+1.96\times \mathrm{stan}\mathrm{dard}\;\mathrm{deviation}\left(\mathrm{SD}\right)\mathrm{of}\;\delta \end{array}$$

(2.2) $${\mathrm{CL}}_{\Delta }\;\left[\%\right]=\left|\mathrm{mean}\;\Delta \right|+1.96\times \mathrm{SD}\;\mathrm{of}\;\Delta$$

To obtain the CL, the mean difference over all repeated measurements for POIs in comparable situations (isocenter vs. nontarget) was calculated. Of note, all POIs except for the isocenter can be classified as nontarget. The deviation (either δ or Δ) for each POI was calculated according to Equations (1.1) or (1.2), respectively. The CL concept was based on the statistics of a normal distribution which expects that 95% of the measured points will result in absolute differences that are lower than the CL.^30^ Both the discrepancies and CL concept were used to judge the performance of the CC algorithm to calculate nontarget doses in a clinically relevant situation. Results were based on a single‐institution experience for one clinical test case (prostate) and evaluated against internationally accepted criteria.^29^, ^30^ Importantly, the random deviations in our approach are mainly attributed to the repetition in measurements spread over multiple weeks. As modern intensity modulated treatments are typically created by the superposition of a large number of field segments, we reasoned that combining the results of our set of POIs (isocenter vs. nontarget) would give an estimate of the CL in clinical practice. Criteria were applied to both IMRT and VMAT since no specific tolerance criteria for the latter technique have been proposed.

The dose outside the PTV is referred to as “nontarget dose”. In this study, two approaches to categorize the nontarget dose, based on AAPM TG 158, were followed.^1^

(1) Nontarget dose can be subdivided into two categories: (a) “in‐field nontarget dose” (i.e., nontarget dose that is within the beam's eye view (BEV) of some segments, such as entrance and exit dose) or (b) “out‐of‐field nontarget dose” (i.e., nontarget dose that is outside the PTV and any BEV—and hence deposited by secondary radiation).^1^ Thus, during treatment delivery a POI may receive its dose both directly through the BEV and from secondary sources.^31^ POI positions were categorized as (a) within the BEV of the computational segment or (b) shielded by MLC and/or orthogonal jaws. Of note, there were 100 and 180 computational segments per beam/arc for IMRT and VMAT, respectively. The secondary radiation in a POI was represented by the relative number of MUs from all segments that do not have the POI under analysis within their BEV.
(2) Nontarget dose can also be classified as one of three approximate dose levels.^1^ In this study, focus was on intermediate (5%−50% of the prescription dose) and low doses (< 5% of the prescription dose).

The dependence of the dose accuracy on distance from the isocenter was also investigated. The isocenter rather than the recommended 50% isodose line was chosen since the definition of field edge in IMRT/VMAT is unclear as compared to conventional radiotherapy.^1^

All *p*‐values < 0.05 were considered statistically significant.

## 3 RESULTS

### 3.1 Microdiamond detector

#### 3.1.1 Angular dependence

**FIGURE 3:**
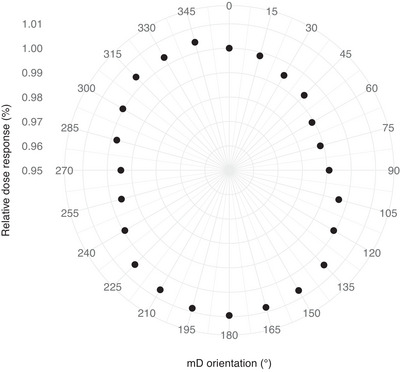
Dose response as a function of mD orientation angle measured in the isocenter. Importantly, the mD was fixed in the detector rod while the rod was manually rotated. Responses were normalized to the response at 0° angle.

The difference between the maximum and minimum value relative to the mean of all values was within 2.0% for both primary field locations (Figure 3). A similar value was obtained for the out‐of‐field location (2.9%). For both primary field locations, the maximum response was observed at 180°, while a maximum increase in response of 2.8% was observed at 210° in the out‐of‐field location. The homogenization of the dose distribution over the mD had no direct effect on the angular dependence, implying that possible nonuniformities in the sensitive volume did not contribute meaningfully.

#### 3.1.2 Positioning and centering method

The longitudinal offset of the mD's EPOM was found to be 1.51 mm towards the gun end. All POIs in RayStation were subsequently shifted accordingly to correct for this offset.

#### 3.1.3 Cable effect

The mean induced extra dose by charge imbalance in the cable in hole 5 was 0.003 Gy ± 0.001 Gy and 0.004 Gy ± 0.001 Gy for IMRT and VMAT, respectively. In hole 9, these values reduced to 0.000 Gy ± 0.001 Gy and 0.001 Gy ± 0.001 Gy, respectively. These values were considerably below the lowest measured doses in any of the specified POIs in hole 5 (0.035 Gy ± 0.002 Gy for IMRT and 0.032 Gy ± 0.001 Gy for VMAT) and hole 9 (0.024 Gy ± 0.002 Gy for IMRT and 0.023 Gy ± 0.001 Gy for VMAT).

### 3.2 Treatment plan measurements

#### 3.2.1 Single static field

All POIs were located in areas where the relative dose level was < 5% of the prescription dose. Local discrepancies (δ) are reported in Table 1. There was a large overestimation of the dose for all POIs located in hole 9 (POIs_1‐5_), both with MLC_0_ and MLC_45_.

**TABLE 1:** δ (%) between calculated and measured nontarget doses for the static field experiments.

				MLC_0_		MLC_45_		MLC_45 with jaws retracted_	
POI^a^	Measurement hole^b^	Longitudinal position^c^ (cm)	Distance to isocenter^d^ (cm)	Relative dose level^e^	δ	Relative dose level^e^	δ	Relative dose level^e^	δ
1	9	−4	19.3	< 1%	58.6%	< 1%	−85.7%	< 1%	54.1%
2	9	0	18.9	< 1%	59.8%	< 1%	−76.3%	< 1%	69.1%
3	9	−2	19.0	< 1%	62.9%	< 1%	−73.2%	< 1%	69.1%
4	9	+2	19.0	< 1%	56.8%	< 1%	−55.3%	< 1%	90.4%
5	9	+4	19.3	< 1%	62.2%	< 1%	−53.7%	< 1%	91.4%
6	7	−6	13.6	[1%−5%]	−20.4%	[1%−5%]	−33.6%	[1%−5%]	−15.2%
7	8	−4	12.0	[1%−5%]	20.4%	[1%−5%]	−22.5%	[1%−5%]	11.9%
8	8	−2	11.5	[1%−5%]	16.6%	[1%−5%]	−22.0%	[1%−5%]	8.8%
9	8	0	11.4	[1%−5%]	11.7%	[1%−5%]	−20.4%	[1%−5%]	8.1%
10	7	−	12.4	[1%−5%]	−16.1%	[1%−5%]	−19.6%	[1%−5%]	−16.3%
11	7	0	12.2	[1%−5%]	−15.8%	[1%−5%]	−17.7%	[1%−5%]	−17.5%
12	7	−4	12.8	[1%−5%]	−10.1%	[1%−5%]	−17.0%	[1%−5%]	−3.1%
13	7	+2	12.4	[1%−5%]	−17.5%	[1%−5%]	−12.3%	[1%−5%]	−18.2%
14	5	+4	7.7	[1%−5%]	−8.9%	[1%−5%]	−3.8%	[1%−5%]	−4.9%
15	5	−4	7.7	[1%−5%]	−6.9%	[1%−5%]	−3.4%	[1%−5%]	−3.1%
16	5	+6	8.9	[1%−5%]	−11.5%	[1%−5%]	−3.2%	[1%−5%]	−4.5%
17	5	0	6.5	[1%−5%]	−8.6%	[1%−5%]	−2.9%	[1%−5%]	−4.6%
18	5	−2	6.8	[1%−5%]	−7.3%	[1%−5%]	−1.8%	[1%−5%]	−4.8%
19	5	+2	6.8	[1%−5%]	−8.5%	[1%−5%]	−1.2%	[1%−5%]	−2.9%
20	7	+4	12.8	[1%−5%]	−13.2%	[1%−5%]	4.4%	[1%−5%]	−3.1%
21	8	+2	11.5	[1%−5%]	12.9%	[1%−5%]	9.0%	[1%−5%]	9.3%
22	8	+4	12.0	[1%−5%]	15.1%	[1%−5%]	22.2%	[1%−5%]	17.0%

*Note*: Dose calculations were done using a 2 × 2 × 2 mm^3^ grid size.

^a^ See also Figure S2.

^b^ See also Figure 1.

^c^ Longitudinal position in the cranial (+) or caudal (−) direction.

^d^ Distance to the isocenter in three dimensions.

^e^ Dose levels are relative to the prescription dose.

**FIGURE 4:**
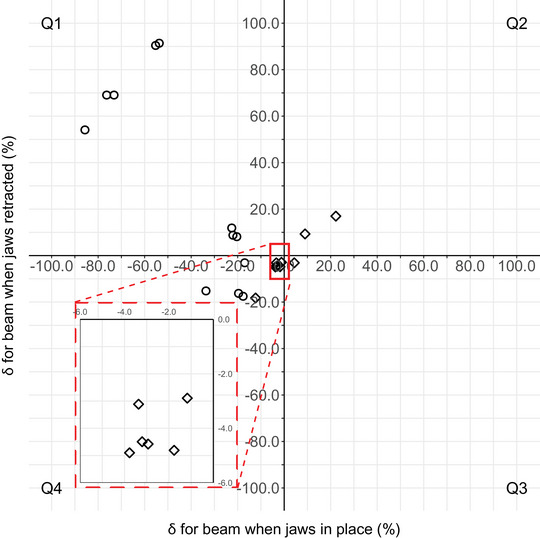
Comparison of δ for a static field with MLC at 45° when jaws were in place (MLC_45_) or retracted (MLC_45 with jaws retracted_). ○ = POIs that were located beneath the orthogonal jaws when these were in place, but were only shielded by the MLC when the orthogonal jaws were retracted (POIs_1‐12_); ◇ = POIs that were shielded by only the MLC during both experiments (POIs_13‐22_). The inset graph shows a close up of the outlined region. Q = quadrant.

The mean δ, averaged over all nontarget POIs, for MLC_0_ (10.5% ± 29.9%) and MLC_45 with jaws retracted_ were significantly different (15.0% ± 35.1%) (*p*‐value < 0.05). Importantly, all POIs experienced only shielding by the MLC. For MLC_45_, however, the TPS underestimated the calculated doses for on average by −22.3% ± 29.2% (*p*‐value = 0.04) as compared to MLC_45 with jaws retracted_ (Figure 4). In contrast, the Δ was within 1% for all cases, independent of the field was collimated by MLC_0_ (range −0.5% to 0.4%), MLC_45_ (range −0.4% to 0.3%) or MLC_45 with jaws retracted_ (range −0.3% to 0.5%).

**FIGURE 5:**
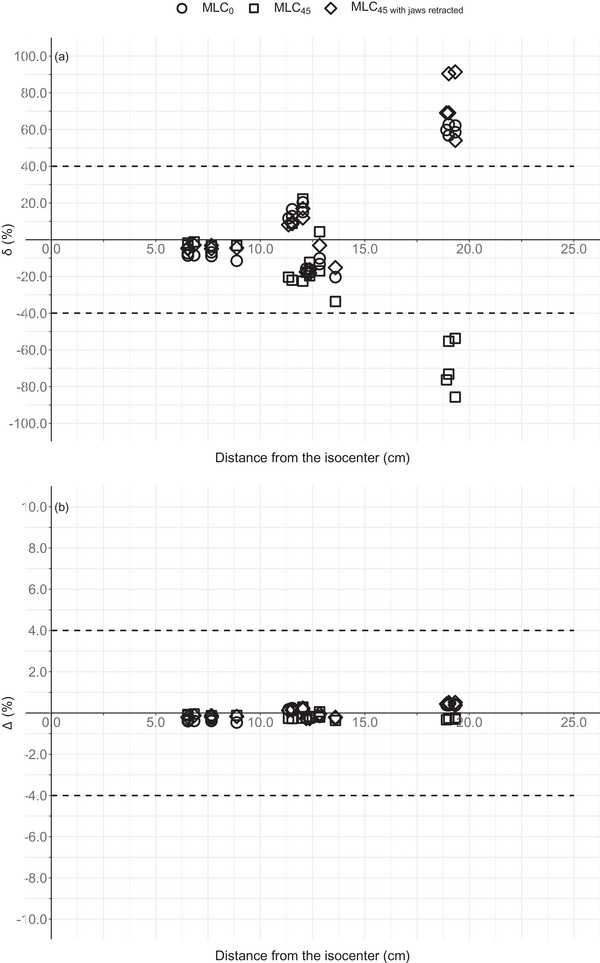
δ (%) (a) and Δ (%) (b) for all nontarget POIs as a function of distance from the isocenter for the single static field experiments. The dashed lines represent the tolerance level as proposed by Venselaar et al.^29^

Figure 5 shows the variation in δ and Δ for all nontarget POIs as a function of distance from the isocenter. For all three plans, δ increased in an approximately exponential way with distance from the isocenter. Up to 15 cm from the isocenter, δ was within the tolerance level (40%).^29^ POIs failing this criteria were located in measurement hole 9 (distance range 18.9–19.3 cm). Moreover, δ was strongly dependent on the position of the orthogonal jaws (in place or retracted).

#### 3.2.2 Prostate radiotherapy

Table 2 summarizes the δ and Δ for the IMRT and VMAT treatment. Dose levels, relative to the prescription dose, ranged from 0.6% to 46.5% for IMRT and 0.6% to 27.3% for VMAT.

**TABLE 2:** Summary of δ (%) and Δ (%) for the IMRT and VMAT prostate treatment plans.

				IMRT				VMAT			
POI^a^	Measurement hole^b^	Longitudinal position^c^ (cm)	Distance to isocenter^d^ (cm)	Secondary radiation^e^	Relative dose level^f^	δ	Δ	Secondary radiation^e^	Relative dose level^f^	δ	Δ
Isocenter	0	0	0.0	48.2%	≥ 50%	1.3%	1.3%	28.8%	≥ 50%	2.2%	2.2%
1	5	+4	7.7	85.7%	[10%−50%]	−10.4%	−2.8%	90.3%	[10%−50%]	−4.7%	−0.9%
2	5	−2	6.8	79.1%	[10%−50%]	−2.0%	−0.8%	83.4%	[10%−50%]	0.8%	0.2%
3	7	0	12.2	77.5%	[10%−50%]	−1.2%	−0.5%	78.8%	[10%−50%]	1.7%	0.5%
4	8	+2	11.5	89.2%	[10%−50%]	2.8%	0.4%	88.4%	[10%−50%]	0.9%	0.2%
5	8	−4	12.0	99.8%	[5%−10%]	−10.2%	−0.6%	78.9%	[5%−10%]	4.6%	0.4%
6	9	+2	19.0	93.2%	[1%−5%]	12.5%	0.6%	94.7%	[10%−50%]	−2.0%	−0.2%
7	9	−4	19.3	89.1%	[5%−10%]	−0.8%	−0.1%	97.1%	[1%−5%]	−6.8%	−0.2%
8	8	+8	13.9	100.0%	[1%−5%]	−31.0%	−0.5%	100.0%	[1%−5%]	−34.8%	−0.6%
9	5	+8	10.3	100.0%	[1%−5%]	−25.7%	−0.5%	100.0%	[1%−5%]	−25.2%	−0.5%
10	5	+7	9.6	100.0%	[1%−5%]	−25.5%	−0.7%	100.0%	[1%−5%]	−22.4%	−0.6%
11	0	+6	6.0	100.0%	[5%−10%]	−25.0%	−1.4%	100.0%	[5%−10%]	−19.8%	−1.1%
12	7	+6	13.6	100.0%	[1%−5%]	−22.3%	−0.6%	99.2%	[1%−5%]	−14.5%	−0.4%
13	5	+6	8.9	100.0%	[1%−5%]	−21.0%	−0.7%	100.0%	[1%−5%]	−15.3%	−0.6%
14	7	−6	13.6	100.0%	[1%−5%]	−15.4%	−0.4%	100.0%	[1%−5%]	−16.2%	−0.4%
15	9	+4	19.3	100.0%	[1%−5%]	17.2%	0.3%	98.4%	[1%−5%]	−23.9%	−1.0%
16	5	+12	13.7	100.0%	< 1%	**−88.1%**	−0.8%	100.0%	< 1%	**−82.6%**	−0.7%
17	9	+10	21.4	100.0%	< 1%	**−54.9%**	−0.3%	100.0%	< 1%	−39.3%	−0.2%

*Note*: Dose calculations were done using a 2 × 2 × 2 mm^3^ grid size. Values are presented in bold if the recommended tolerance values for δ and Δ were exceeded.^29^, ^30^

^a^ POIs listed in this Table do not refer to the POIs reported in Table 1 (See also Figure S3).

^b^ See also Figure 1.

^c^ Longitudinal position in the cranial (+) or caudal (−) direction.

^d^ Distance to the isocenter in three dimensions.

^e^ POIs receiving only secondary dose (100.0%) (see also Figure 5) are classified as “out‐of‐field” following the definition suggested by Kry et al.^1^

^f^ Dose levels are relative to the prescription dose.

δ in the isocenter for IMRT was 1.3%. The obtained CL_δ_ (2.1%) was within the recommended tolerance value (3%).^29^ δ in the nontarget region was −17.7% ± 25.3% (range −88.1% to 17.2%). IMRT failed the recommended criteria (40%)^29^ for the CL_δ_ (68.8%). Δ was −0.5% ± 0.8% (range −2.8% to 0.6%). The corresponding CL_Δ_ was 1.9%, which was below the recommended tolerance value (4%).^29^, ^30^

δ in the isocenter for VMAT was 2.2%, while the δ in the nontarget region was −17.6% ± 21.2% (range −82.6% to 4.6%). VMAT passed the recommended CL_δ_ value in the isocenter (2.7% vs. 3%)^29^ but failed in the nontarget region (52.6% vs. 40%).^29^ The Δ was −0.4% ± 0.5% (range −1.1% to 0.5%). The corresponding CL_Δ_ (1.3%) was in agreement with the recommended tolerance criteria (4%).^29^, ^30^

**FIGURE 6:**
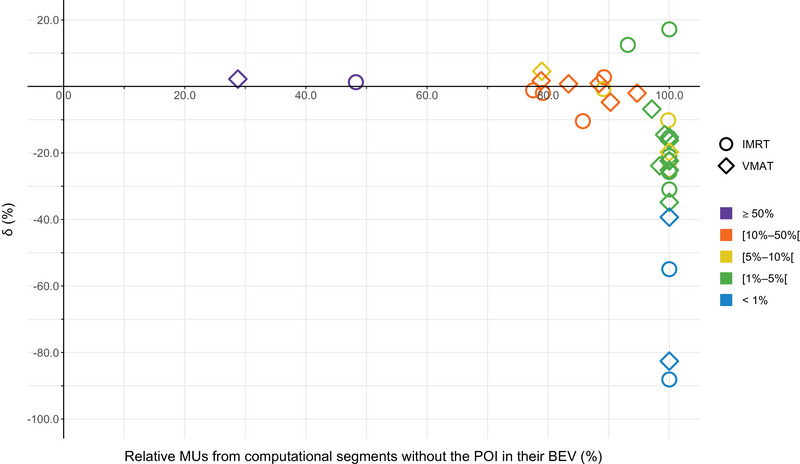
δ (%) for all POIs (including the isocenter) as a function of the relative MUs from computational segments that do not have the POI in their BEV for IMRT and VMAT (x = 0%, all segments irradiate the POI under analysis directly and x = 100%, the POI under analysis receives exclusively secondary dose from all segments). Dose calculations were done using a 2 × 2 × 2 mm^3^ grid size. Symbol colors refer to the dose level relative to the prescription dose.

The relative amount of MUs from computational segments without the POI in their BEV in the isocenter was 48.2% for IMRT and 28.8% for VMAT (Figure 6). For POI_1‐17_, the average relative amount was 94.9% ± 7.9% (range 77.5%–100.0%) for IMRT and 94.7% ± 7.7% (range 78.8%–100.0%) for VMAT. In addition, measured doses for POIs that receive only secondary radiation, and could thus be categorized as out‐of‐field, were significantly different from calculated values (*p*‐value < 0.01 for both IMRT and VMAT). Both lower and higher values were reported. This was in contrast with in‐field POIs (*p*‐value = 0.3 for IMRT and *p*‐value = 0.4 for VMAT). Moreover, the CL_δ_ for out‐of‐field POIs did not satisfy the tolerance criteria (40%)^29^ for both IMRT (85.5%) and VMAT (69.3%). In contrast, CL_δ_ for the in‐field POIs was below the tolerance criteria for IMRT (14.0%) and VMAT (23.6%). The CL_Δ_ was below 4% for both abovementioned subcategories for both techniques.^29^, ^30^

Calculated values were significantly different from measured doses for POIs located below the 5% prescription dose level (*p*‐value < 0.05 for both IMRT and VMAT) as compared to POIs receiving ≥ 5% of the prescription dose (*p*‐value = 0.1 for IMRT and *p*‐value = 0.6 for VMAT). In addition, POIs located in the < 5% dose region were above the CL_δ_ tolerance level (40%)^29^ for both IMRT (88.7%) and VMAT (62.8%). POIs located in the ≥ 5% relative dose region did satisfy this criteria (22.3% for IMRT and 14.2% for VMAT).

**FIGURE 7:**
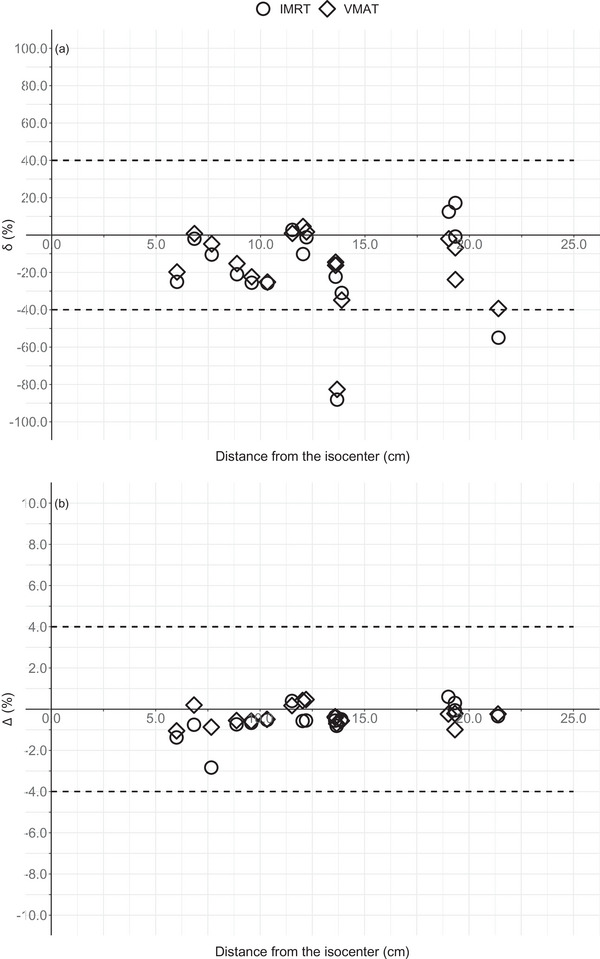
δ (%) (a) and Δ (%) (b) for all nontarget POIs as a function of distance from the isocenter for the prostate treatment plans. The dashed lines represent the tolerance level as proposed by Venselaar et al.^29^

As can be seen in Figure 7, the distance from the isocenter is not a determining factor for δ. The POIs failing the tolerance criteria (40%)^29^ (POI_16_ and POI_17_) were located near the edge of the phantom (1 and 3 cm from the cranial end, respectively), receiving < 1.0% of the prescribed dose. Although the number of MUs was higher for IMRT (906.1 vs. 695.8 for VMAT), which can increase leakage contributions, IMRT and VMAT differed very little in terms of absolute doses in the POIs (either calculated [*p*‐value = 0.7] or measured [*p*‐value = 0.7]), and in terms of δ (*p*‐value = 0.3) or Δ (*p*‐value = 0.2).

**FIGURE 8:**
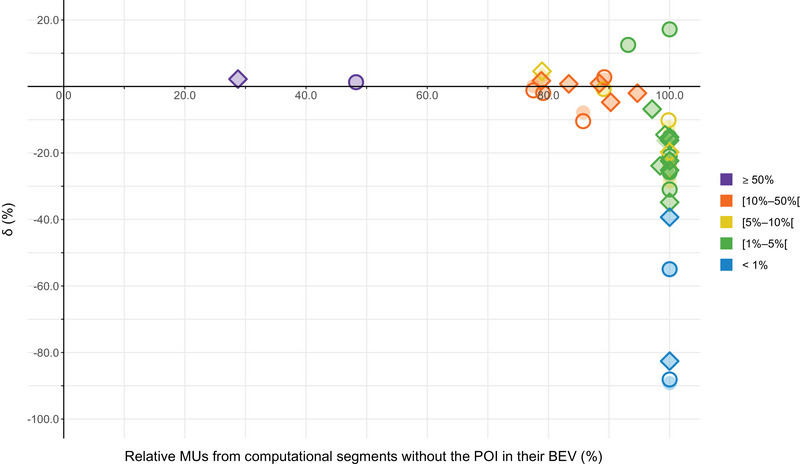
Comparison of δ (%) for all POIs (including the isocenter) as a function of the relative MUs from computational segments that do not have the POI in their BEV for IMRT and VMAT between the different grid sizes. Symbol colors refer to the dose level relative to the prescription dose. Open symbols = grid size 2 × 2 × 2 mm^3^ and filled symbols = 1 × 1 × 1 mm^3^. ○ = IMRT and ◇ = VMAT.

The effect of grid size in the TPS was also evaluated (Table S1). The maximum absolute dose discrepancy was 0.027 Gy (POI_1_) and 0.006 Gy (POI_11_) for IMRT and VMAT, respectively (Figure S5). δ between both grid sizes were comparable (Figure 8). A visual voxel‐by‐voxel comparison of the calculated doses showed that dose agreement was worse in regions with a high dose gradient (e.g., for rectum sparing or rapid dose fall‐off around the target). Overall, a good dose agreement was seen in the low‐dose gradient regions. Since no POIs were located in high‐dose gradient regions — except POI_1_, which was located in dose gradient 3.6% mm^−1^ (relative to the prescription dose of 56 Gy) during IMRT — a voxel dimension of 2 × 2 × 2 mm^3^ produced acceptable results without substantially compromising accuracy. Moreover, upon careful review of the results, the impact of the grid size on our conclusions was marginal.

## 4 DISCUSSION

Although nontarget doses are rarely known adequately, they are of interest when there are OARs in close proximity to the primary target. Here, the nontarget dose calculation accuracy and reliability of the CC algorithm implemented in the RayStation TPS was evaluated by directly comparing point measurements with calculations using a pelvic phantom and CT‐based (patient) images.

For the single static fields experiments, discrepancies between calculations and measurements typically depended upon whether the jaws were in place or retracted. POIs that were shielded by only the MLC in both experiments (POIs_13‐22_ with symbol ◇ in Figure 4) were, as expected, more or less following the 45° diagonal in quadrants Q2 and Q4. In contrast, doses for POIs_1‐12_ were either underestimated due to the modelling of the orthogonal jaws as perfect attenuators in RayStation or overestimated, on the average, due to a relatively high leaf transmission parameter value (see further).

Although the CL_δ_ in the nontarget regions failed the proposed tolerance value for both IMRT and VMAT, the CL_Δ_ was within the tolerance criteria. No action limits (7%)^30^ were exceeded. Overall, the nontarget accuracy was comparable between the different techniques because the same fundamental limitations in TPSs exist.^32^

Results of our prostate treatments (Table 2) are in agreement with previously reported results for an IMRT prostate plan, where underestimations were observed beyond the 5% isodose and over‐ and underestimations were reported between the 50% and the 5% isodose in comparison with EBT3 film.^33^ In this work, POIs located below the 5% relative dose level were significantly underestimated by the TPS for both IMRT and VMAT, except for POI_15_ during the IMRT treatment (δ = 17.2%). In addition, a POI is located, for a specific computational segment, inside or outside the BEV, with its position possibly changing within the course of the same treatment delivery. Since modern modulated treatment plans are typically characterized by a high number of MUs, a significant fraction of the dose to points outside the primary target may come from secondary sources.^1^, ^31^, ^34^ This is illustrated in Figure 6, where the local dose accuracy is lowest for POIs that are rarely within the BEV and receive a high amount of secondary radiation. This is basically similar to the observation of Schwarz et al.^35^ In this study, POI_16_ and POI_17_ were the most challenging points, both located in the < 1% dose region and receiving only secondary radiation. Such POIs can push the CC algorithm to its limits. Excluding these POIs improved the CL_δ_ for IMRT (36.9%) and VMAT (33.0%), now both satisfying the recommended criteria.

Figures 5 and 7 show the discrepancies as a function of the distance from the isocenter. Overall, the TPS could be considered as acceptably accurate up to 15 cm from the isocenter. For the single static field experiments, accuracy was indeed strongly dependent on distance from the isocenter, in accordance with other research results.^4^ For prostate treatment plans, other factors such as the planning experience of the dosimetrist or applied class solution are of more importance.

The authors propose several physical explanations and identify some sources of error for the observed discrepancies. First, the Agility™ orthogonal collimator includes a single pair of sculpted jaws, which have a Y‐shaped zone with a high (jaw_thick_) and low attenuation area (jaw_thin_).^36^ While the critical parameter of jaw transmission is explicitly taken into account in other TPSs,^37^ the orthogonal jaws are assumed to have zero transmission in RayStation.^38^ However, maximum transmission values at 6 MV of 0.07%, 0.13%, and 0.32% have been measured for areas shielded by MLC + jaw_thick_, MLC + jaw_thin_ and jaw_thick_, respectively.^36^ This explains why in Figure 4, the majority of POIs are situated in Q1 and Q4. Thus, differences can be dosimetrically magnified by their non‐physical modelling of the jaws in the TPS.

Secondly, leaf transmission is usually experimentally determined and specified by the user. Only an average value, based on the interleaf and intraleaf leakage, is used as input value in RayStation.^38^ Our leaf transmission model value for a 6 MV photon beam on an Elekta Synergy equipped with an Agility™ collimator (0.0055) is comparable with some studies,^39^, ^40^ but higher than other reported values.^36^, ^41^

**FIGURE 9:**
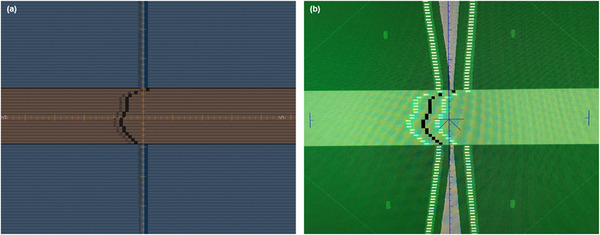
Typical leaf behavior behind the jaws for an IMRT treatment beam in (a) the TPS and (b) the TCS during delivery. The actual position of the leaves behind the diaphragm in dynamic photon mode is following a fan shape, which is not modelled by RayStation.

Furthermore, there is an essential difference in leaf behavior behind the jaws in RayStation versus the TCS, both in static and dynamic mode.^42^ During the single static field experiments, leaves behind the jaws are positioned so that there is a gap between opposing leaves, irrespective of their prescribed positions. During IMRT and VMAT, the actual position of the leaves behind the jaws depends on the prescribed position and the distance from the leading edge of the jaw. The first two leaves outside of the field are determined by the TPS and R&V system. Subsequent leaf are positioned following the shape of a fan (Figure 9). Since this is not a usual prescription from the TPS, this leaf behavior is not taken into account during dose calculations and can lead to further dose underestimations for POIs located within the fan shape.

In addition, the CC algorithm in RayStation aligns all point spread kernels with the CAX.^43^ This contradicts the reality that energy propagation, including scatter, at field edges is not parallel to the CAX, resulting in an underestimation of the patient scatter component. Other potential sources of error are the incorrect modelling of output factors for small fields and the inaccurate positioning of the leaves/jaws.^44^ With regard to the MLC position, RayStation models the MLC offset using a leaf‐tip offset rather than a dosimetric leaf gap.^38^ Previous work showed that changes in MLC tip offset can produce dose differences up to 25% in OARs.^45^ Thus, this parameter can have a substantial impact on the accuracy of nontarget dose calculations for modulated treatments by a commercial TPS. This work supports previous recommendations^33^ to develop new dedicated analytical models for accurate nontarget dose predictions.

To put the measured discrepancies into clinical perspective, we calculated the absolute dose error for POIs located in a clinically relevant situation (POI_4‐5_ and POI_8‐11_ in Table 2) for a complete treatment schedule with 16 fractions. The dose errors ranged from 20.6 to 62.1 cGy for IMRT and 10.6 to 51.4 cGy for VMAT. Although the clinical impact of these doses has yet to be fully disclosed, previous studies have demonstrated that even low radiation doses may reshape the immune system and gastrointestinal microbiota.^46^, ^47^ These are two systems that are hypothesized to modulate the clinical response to ionizing radiation.^7^, ^48^ In addition, good evidence of an increase in some cancer risks has been shown for fractionated exposure to doses > 100 mSv, and valid evidence for an increase in risk at doses ≥ 50 mSv.^49^ Thus, the reported doses in this study agree with previous results.

Nontarget dosimetry is challenging due to the non‐standard radiation conditions the detectors are exposed to in these regions. During modern intensity modulated treatments the energy spectrum as well as the incident beam angle in a specific POI are continuously varying, making it nearly unfeasible to correctly compensate the detector response for those discrepancies.^50^ Therefore, the detector selection and characterization is of utmost importance. The angle dependence of the mD detector was within 2.0% and 2.9% for primary field and out‐of‐field locations, respectively. These results were similar to previously reported findings that the mD had an nearly angular independent response for field sizes ≥ 2 × 2 cm^2^ (maximum variation of 2%).^19^ Other studies reported an angular fluctuation < 1.0%.^13^, ^17^ Overall, the angle dependence of the mD detector can be considered negligible. In addition, there was an insignificant cable effect in agreement with Brace et al., who concluded that the cable is a minor contributor to the extra cameral effect of the mD detector.^19^

This work is limited because the results are specific to the RayStation v6 TPS and an Elekta linac equipped with an Agility™ collimator. In addition, results are based on a single‐institutional experience for one clinical test case (prostate). Although it is useful to evaluate nontarget dose calculations in a homogeneous water phantom, the accuracy of a TPS in these regions should ideally also be validated for more real clinical cases, for example, by using a heterogeneous phantom equipped with different material densities as this would impose additional dose calculation challenges to the TPS. Indeed, differences in electron density from water will introduce extra uncertainties.^51^ However, the approach used in this work revealed several effects (see also Figures 4 and 9) that will persist. It was also not verified whether adjusting specific modelling parameters could improve the performance of our TPS as this was beyond the scope of this research. However, dose recalculation in the same measurement points using an alternative clinical beam model in the Pinnacle^3^ (v9.0) TPS did not significantly improve the overall accuracy of the out‐of‐field dose calculations in the study of Huang et al.^32^ A dose grid resolution of 2 mm/voxel produced acceptable results without substantially compromising accuracy (Figure 8).

## 5 CONCLUSION

This study provides data about the level of dose calculation accuracy and reliability that can be expected for modern prostate radiotherapy in nontarget regions with the CC dose calculation algorithm implemented in the RayStation TPS. In general, nontarget doses for prostate treatments were underestimated by as much as −88.1% (δ) in regions where the relative dose level was < 5% of the prescribed dose. In higher relative dose regions (≥ 5%) both over‐ and underestimations were seen. In addition, the accuracy was also steered by the contribution from secondary sources. The dose in POIs located in such low dose regions or receiving a high amount of secondary radiation was poorly predicted by the CC algorithm. Since the measured and calculated doses differed no more than the recommended action level, our TPS can be considered acceptable for clinical use.

Improving dosimetric accuracy is challenging due to approximations inherent to the CC algorithm. In addition, the current non‐physical assumptions in the TPS of the MLC delivery model and orthogonal jaw transmission hinder an accurate beam commissioning in nontarget regions. Of course, any clinical TPS and inherent beam modeling is subject to a reasonable trade‐off between accuracy and computational efficiency, especially during IMRT or VMAT optimization. A separate or incorporated dose calculation algorithm that can accurately compute nontarget radiation doses could be of great value in the study of the clinical impact of these low doses.

The primary goal of this work was to report on the dosimetric accuracy of nontarget doses made by a commercial TPS. Although some results were out of the mentioned tolerance levels, it is still the responsibility of the treating radiation oncologists or researchers to decide whether these inaccuracies are of relevance.

## AUTHOR CONTRIBUTIONS

All authors have made substantial contributions either to the conception and design of the study, to the collection and assembly of the data or to the interpretation of the results. All authors have been involved either in drafting the manuscript or in revising it critically.

## CONFLICT OF INTEREST STATEMENT

No conflicts of interest.

## Supporting information

Supporting Information
