# Supplementary material for: Accuracy and reliability of a commercial treatment planning system in nontarget regions in modern prostate radiotherapy
Source: J Appl Clin Med Phys. 2023 May 11;24(8):e14003. doi: 10.1002/acm2.14003 (PMC10402685; doi:10.1002/acm2.14003)
Supplement: Supplementary file 1 — Supporting Information [file ACM2-24-e14003-s001.pdf]

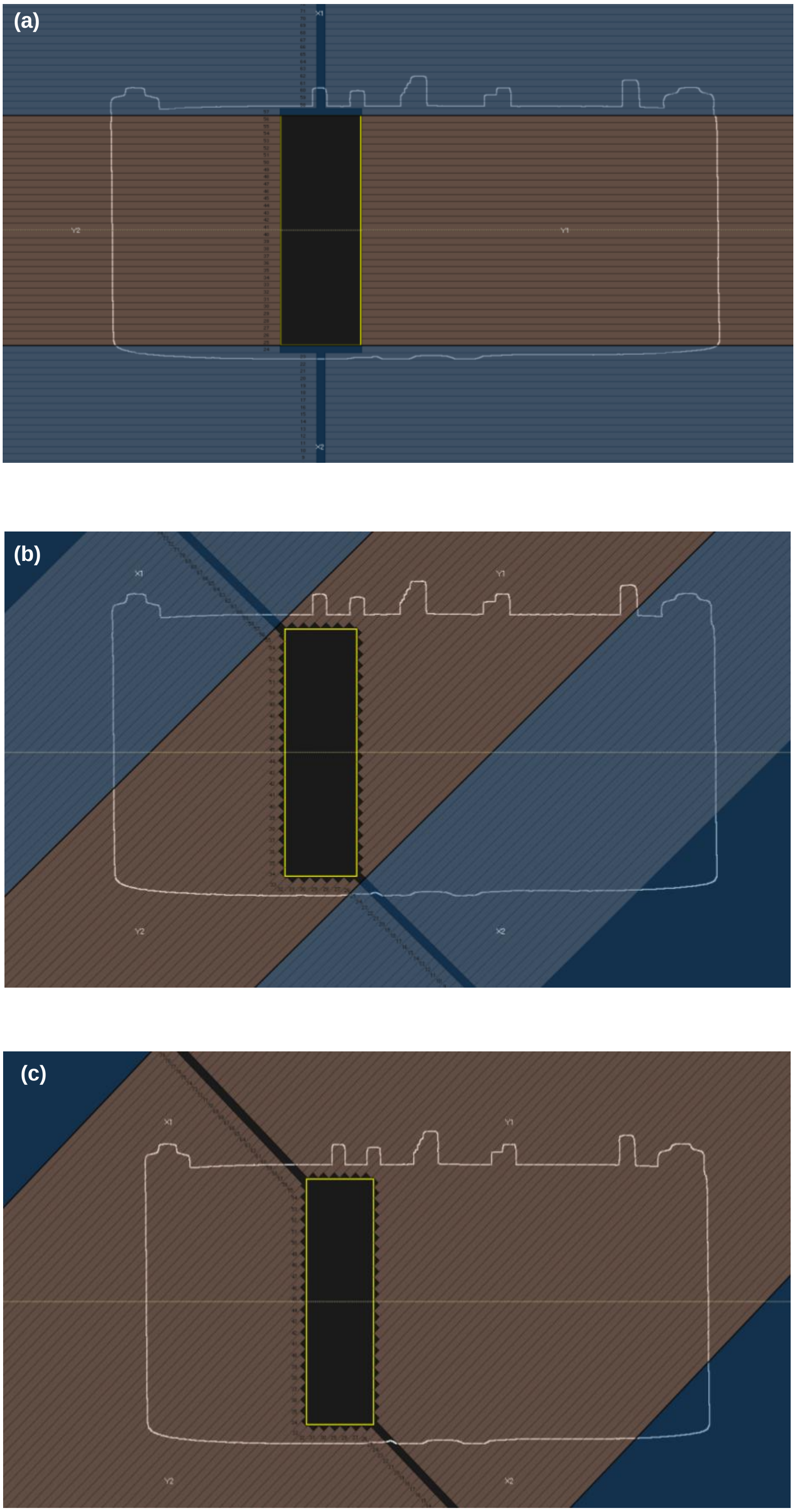

Fig. S1 BEV of the single static fields: (a) MLC_0; (b) MLC_{45}; and (c) $MLC_{45\ \text{with jaws retracted}}$. The pelvic phantom is delineated in white, while the PTV_a is contoured in yellow. Jaws and MLC leaves are shown in blue and brown, respectively. Jaws are labelled according to the IEC 601 standard.

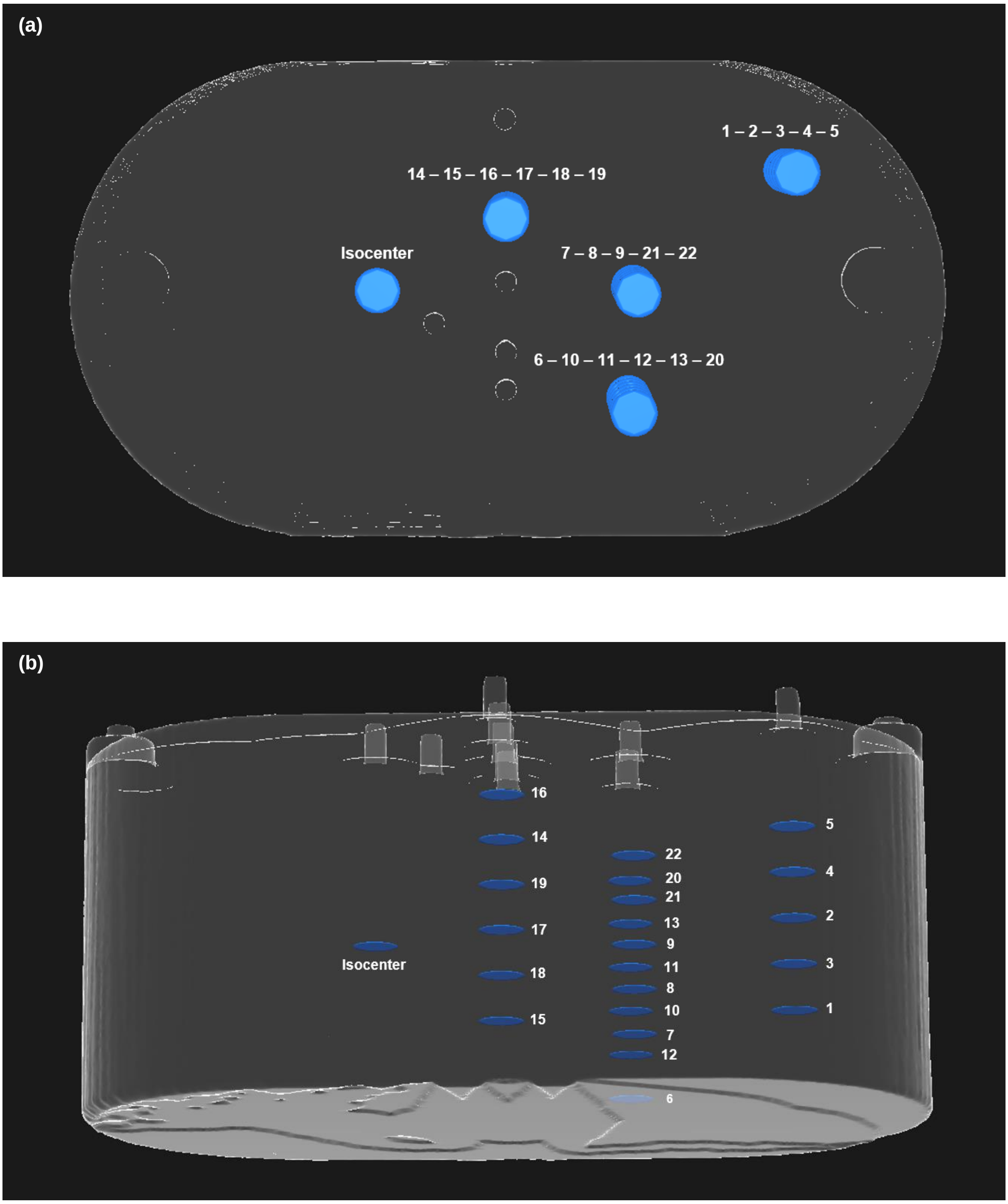

Fig. S2 Transverse view (a) and coronal perspective (b) of the pelvic phantom. The location of each POI (see Table 1) is indicated by a blue disk, representing the mD in measurement position (not to scale). All POIs were defined for the three experimental setups with a single static field. Within the TPS, point doses at the equivalent location were determined using the point dose tool.

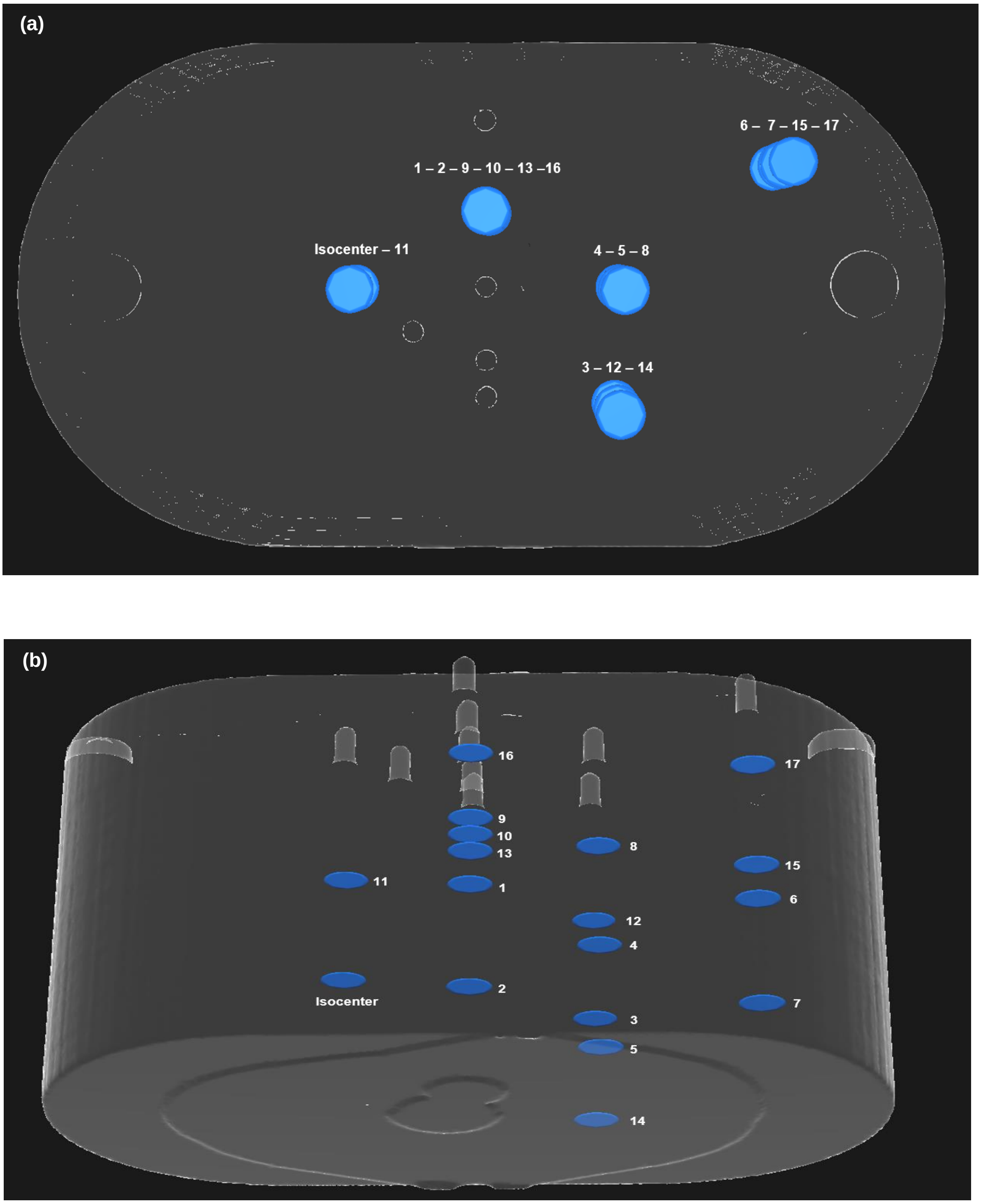

Fig. S3 Transverse view (a) and coronal perspective (b) of the pelvic phantom. The location of each POI (see Table 2 and Supplementary Table S1) for the prostate treatment plans is indicated by a blue disk, representing the mD in measurement position (not to scale). Within the TPS, point doses at the equivalent location were determined using the point dose tool.

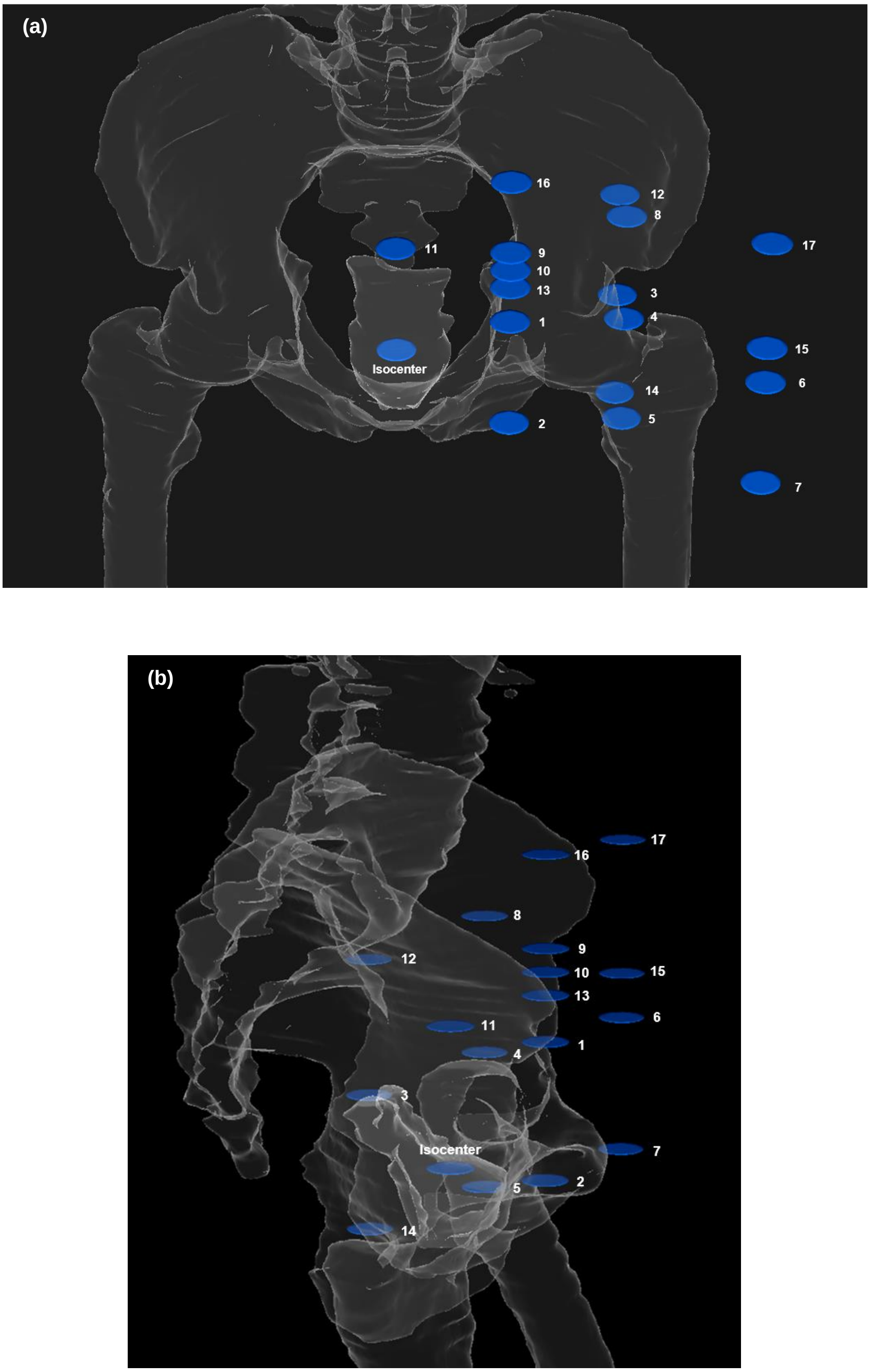

Fig. S4 Coronal (a) and sagittal (b) perspective of the 18 POIs mapped on the prostate cancer patient. The location of each POI (see Table 2 and Supplementary Table S1) is indicated by a blue disk, representing the mD in measurement position (not to scale). The CTV and bony anatomy of the patient are displayed as reference points.

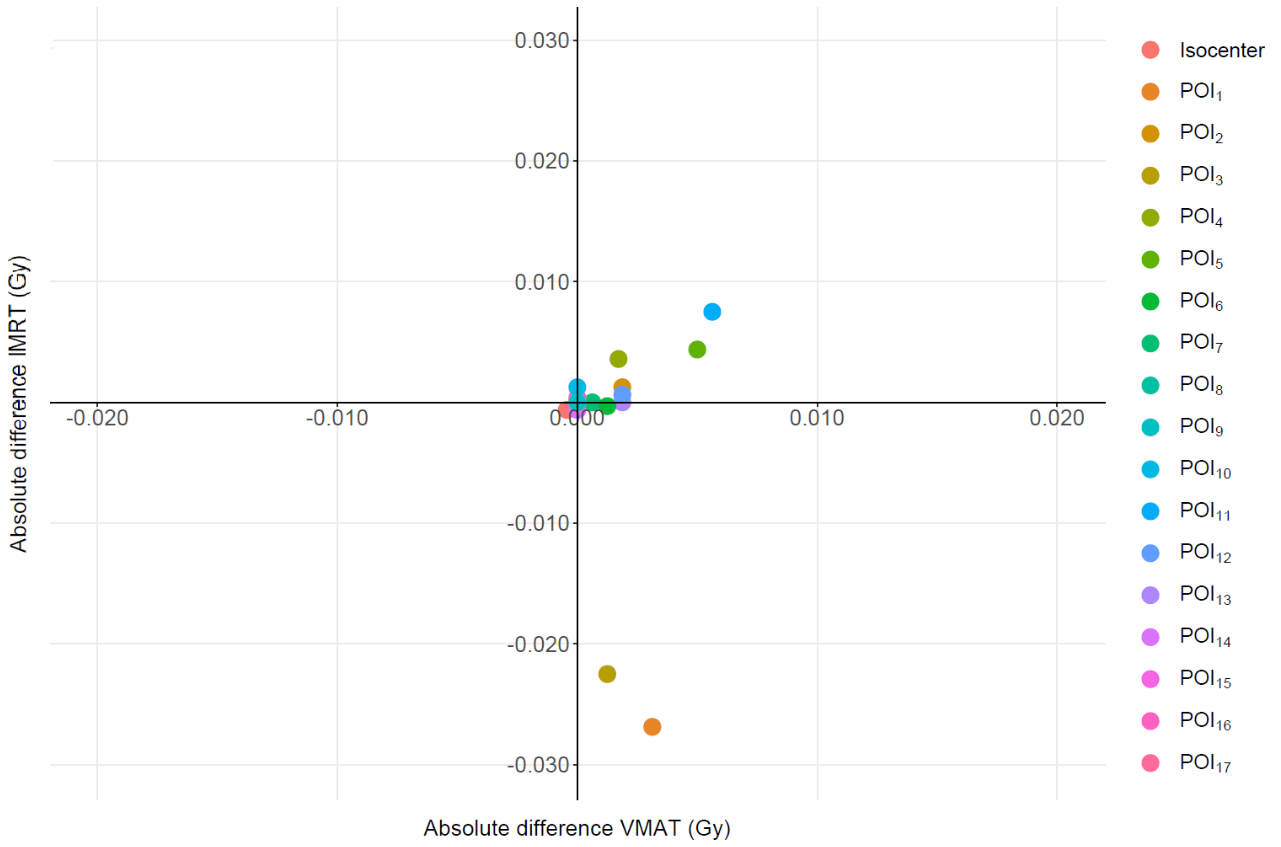

Fig. S5 Absolute dose differences between grid sizes 2 × 2 × 2 mm³ and 1 × 1 × 1 mm³ for IMRT and VMAT. The dose grid resolution of 2 mm/voxel was considered as reference.

Supplementary Table S1 Summary of δ (%) and Δ (%) for the IMRT and VMAT prostate treatment plans. Dose calculations were done using a 1 × 1 × 1 mm³ grid size. Values are presented in bold if the recommended tolerance values for δ and Δ were exceeded[29,30].

				IMRT				VMAT			
**POI[a]**	**Measurement hole[b]**	**Longitudinal position[c] (cm)**	**Distance to isocenter[d] (cm)**	**Secondary radiation[e]**	**Relative dose level[f]**	**δ**	**Δ**	**Secondary radiation[e]**	**Relative dose level[f]**	**δ**	**Δ**
Isocenter	0	0	0.0	48.2%	≥ 50%	1.3%	1.3%	28.8%	≥ 50%	2.2%	2.2%
1	5	+4	7.7	85.7%	[10%–50%[	-7.8%	-2.1%	90.3%	[10%–50%[	-5.2%	-1.0%
2	5	-2	6.8	79.1%	[10%–50%[	-2.0%	-0.8%	83.4%	[10%–50%[	0.6%	0.2%
3	7	0	12.2	77.5%	[10%–50%[	0.1%	0.0%	78.8%	[10%–50%[	1.6%	0.4%
4	8	+2	11.5	89.2%	[10%–50%[	2.1%	0.3%	88.4%	[10%–50%[	0.7%	0.1%
5	8	-4	12.0	99.8%	[5%–10%[	-12.2%	-0.7%	78.9%	[5%–10%[	3.1%	0.3%
6	9	+2	19.0	93.2%	[1%–5%[	12.7%	0.6%	94.7%	[10%–50%[	-2.3%	-0.3%
7	9	-4	19.3	89.1%	[5%–10%[	-0.8%	-0.1%	97.1%	[1%–5%[	-7.3%	-0.2%
8	8	+8	13.9	100.0%	[1%–5%[	-31.0%	-0.5%	100.0%	[1%–5%[	-34.8%	-0.6%
9	5	+8	10.3	100.0%	[1%–5%[	-25.7%	-0.5%	100.0%	[1%–5%[	-25.2%	-0.5%
10	5	+7	9.6	100.0%	[1%–5%[	-26.8%	-0.7%	100.0%	[1%–5%[	-22.4%	-0.6%
11	0	+6	6.0	100.0%	[5%–10%[	-28.7%	-1.6%	100.0%	[5%–10%[	-22.5%	-1.2%
12	7	+6	13.6	100.0%	[1%–5%[	-23.0%	-0.6%	99.2%	[1%–5%[	-16.3%	-0.5%
13	5	+6	8.9	100.0%	[1%–5%[	-21.0%	-0.7%	100.0%	[1%–5%[	-16.6%	-0.6%
14	7	-6	13.6	100.0%	[1%–5%[	-14.7%	-0.3%	100.0%	[1%–5%[	-16.2%	-0.4%
15	9	+4	19.3	100.0%	[1%–5%[	17.2%	0.3%	98.4%	[1%–5%[	-23.9%	-1.0%
16	5	+12	13.7	100.0%	< 1%	**-89.2%**	-0.8%	100.0%	< 1%	**-82.6%**	-0.7%
17	9	+10	21.4	100.0%	< 1%	**-54.9%**	-0.3%	100.0%	< 1%	-39.3%	-0.2%

[a] POIs listed in this Table do not refer to the POIs reported in Table 1 (See also Supplementary Fig. S3).
[b] See also Fig. 1.
[c] Longitudinal position in the cranial (+) or caudal (-) direction.
[d] Distance to the isocenter in three dimensions.
[e] POIs receiving only secondary dose (100.0%) (see also Fig. 8) are classified as “out-of-field” following the definition suggested by Kry et al[1].
[f] Dose levels are relative to the prescription dose.
